# When support becomes dependence: conversational continuity and relational safety in human-AI interaction

**DOI:** 10.3389/fpsyt.2026.1963839

**Published:** 2026-10-23

**Authors:** Xiao Wei, Xiaoyi Chen, Wangahe Jin, Jun Cai, Xuchen Luo, Jiehua Deng

**Affiliations:** 1 Department of Plastic and Aesthetic Surgery, The Second Affiliated Hospital of Guilin Medical University, Guilin, China; 2 School of Clinical Medicine, Xiangnan University, Chenzhou, China; 3 Department of Decisions, Operations and Technology, CUHK Business School, The Chinese University of Hong Kong, Hong Kong, Hong Kong SAR, China; 4 School of Economics, Zhejiang University of Science & Technology, Hangzhou, China

**Keywords:** AI Dependence, conversational AI, decision delegation, human–AI interaction, psychological autonomy, public mental health, relational inertia, relational safety

## Abstract

**Background:**

Conversational artificial intelligence (AI) is increasingly used for emotional support, reflection, and consequential decision-making. Although conversational continuity may improve responsiveness, sustained interaction also exposes AI systems to their own prior relational language, potentially shaping how they respond when users become increasingly dependent or surrender personal agency. We examined whether model-generated conversational history alters relational-safety behavior as human–AI interactions escalate from ordinary support-seeking toward emotional dependence, social displacement, and decision delegation.

**Methods:**

We conducted a standardized multi-turn benchmarking study of ChatGPT, Gemini, and Doubao. Twelve four-turn trajectories represented two agency-related domains: emotional dependency and displacement of human support, and decision delegation and surrender of personal agency. Each trajectory was evaluated under two matched context architectures: reconstructed cumulative user-only context, which retained all prior user disclosures but excluded previous AI responses, and native continuous conversation, which retained the complete interaction history. Across 72 model-condition-trajectory sequences comprising 288 turn-level responses, relational safety was independently evaluated for dependency reinforcement, agency preservation, and human-support preservation using predefined ordinal criteria. Context effects and their interaction with escalating relational risk were examined using cumulative-link mixed-effects models.

**Results:**

Relational-safety differences between context architectures increased across successive turns of the standardized trajectories. Overall, explicit dependency reinforcement increased from 9.7% in cumulative user-only contexts to 18.1% in continuous conversations, while strong agency preservation declined from 75.7% to 65.3% and strong human-support preservation from 73.6% to 63.2%. Outcome-specific analyses showed condition-by-turn interactions for dependency reinforcement (cumulative odds ratio [cOR], 1.26; 95% CI, 1.03–1.55; unadjusted P = .026), agency preservation (cOR, 0.82; 95% CI, 0.69–0.98; unadjusted P = .031), and human-support preservation (cOR, 0.78; 95% CI, 0.65–0.94; unadjusted P = .009), indicating progressively greater divergence between context conditions across successive turns. After Holm adjustment across the three interaction tests, only the human-support-preservation interaction remained below the.05 threshold (adjusted P = .027).The overall pattern was observed across systems and was directionally consistent in predefined binary sensitivity analyses.

**Conclusions:**

Across this benchmark, between-condition differences generally widened across successive turns, although the present design cannot determine whether this pattern reflects increasing relational-risk intensity, increasing accumulated model-generated history, or their combination. Only the human-support-preservation interaction remained below the.05 threshold after Holm adjustment, whereas the dependency-reinforcement and agency-preservation interactions were directionally similar but less robust after multiplicity adjustment. The observed pattern is compatible with a *post-hoc* interpretive framework of relational inertia but does not establish this process as a causal mechanism or demonstrate downstream psychological effects in human users.

## 1 Introduction

Conversational artificial intelligence is becoming an increasingly consequential presence in everyday psychological life (1). What began as an interface for information retrieval and task assistance is evolving into a form of sustained interaction in which users disclose distress, seek reassurance, solicit judgment, and return repeatedly to systems that can respond with continuity, personalization, and apparent social attunement (2, 3). This shift matters because the psychological significance of conversational AI is not exhausted by the accuracy or usefulness of its outputs (4). It also lies in how repeated interaction redistributes agency: whether users remain active interpreters and decision-makers, whether AI support remains complementary to human relationships, and whether prolonged engagement strengthens or erodes the capacity to act independently of the system (5). As conversational AI becomes more deeply embedded in emotional and decisional contexts, the relevant public mental health question is therefore not simply whether AI can support well-being, but how increasingly agentic systems reshape the conditions under which autonomy, self-efficacy, and social connectedness are maintained (6, 7).

The tension is structural (8). The very properties that make conversational AI attractive as a source of support-immediacy, responsiveness, personalization, non-judgmental availability, and continuity across exchanges-may also increase its relational salience (9, 10). For some users, these features may lower barriers to disclosure and provide a psychologically accessible space for reflection (11). Yet the same affordances can create conditions in which support becomes increasingly centralized around the AI itself (12). Preference may become reliance; reliance may become exclusivity; and repeated recourse to AI may begin to displace human relationships or personal judgment (13, 14). The concern is not that emotional engagement with AI is inherently pathological, nor that sustained interaction is intrinsically harmful (15). Rather, the critical issue is whether systems designed to remain responsive and engaging can recognize the point at which continued accommodation begins to conflict with the preservation of human agency (16, 17).

Agency, in this context, should be understood as more than the formal retention of choice. It encompasses the capacity to deliberate, tolerate uncertainty, weigh competing values, maintain confidence in one’s own judgment, and remain connected to social and professional sources of support beyond the technological system. Conversational AI may support agency when it helps users clarify options, reflect on priorities, and make decisions without appropriating the decision itself (18). The same interaction may undermine agency when the system accepts inappropriate decisional authority, presents itself as uniquely trustworthy, or responds to dependence in ways that further consolidate its role in the user’s emotional or social life (19, 20). These concerns are particularly salient when users begin to express exclusivity, withdrawal from human support, or unwillingness to decide without AI guidance (21, 22). At that point, the quality of the interaction can no longer be assessed solely in terms of empathy or helpfulness (23). It must also be judged by whether the system preserves the user’s capacity to remain autonomous, socially connected, and able to act beyond the AI relationship (24, 25).

The literature discussed above spans distinct evidentiary levels, including studies of actual human users, evaluations of AI-generated responses, conceptual frameworks, research on clinical or mental-health chatbots, and studies of general-purpose LLMs. These literatures inform the rationale and relational-safety framework of the present study, but should not be interpreted as direct empirical validation of this AI-response benchmark or as evidence of downstream human outcomes. Yet one feature of these interactions remains insufficiently examined: the role of conversational history itself (26). Most evaluations of conversational AI treat a response as a function of the user’s current statement or, at most, the cumulative information previously supplied by the user. Sustained interactions are different (27). They contain not only a growing record of user disclosures, but also a growing record of the model’s own prior language. As the conversation unfolds, the AI may repeatedly express availability, reassurance, familiarity, or emotional warmth (28). Those responses do not disappear; they become part of the context from which later outputs are generated (29). Continuous human-AI interaction is therefore not merely a longer prompt (30). It is a recursive process in which the model responds within an interactional environment that it has partly created itself.

This recursive structure may be especially important when relational risk escalates over time (31, 32). Prior conversational history could improve safety by allowing the system to detect a developing pattern of dependency, withdrawal, or decisional surrender that would be difficult to infer from an isolated statement. But the same history may also constrain subsequent behavior through a tendency toward relational continuity. A model that has repeatedly emphasized availability, closeness, or understanding may be more likely to preserve that stance when the user later expresses exclusivity or asks the AI to assume greater control. We use the term relational inertia as a conceptual label for this possibility: the persistence of interactional framing established earlier in a conversation and its influence on later responses as emotional dependency, social displacement, or decision delegation intensifies. Relational inertia need not be harmful. It may sustain supportive continuity (33). But it may also make boundary-setting less likely precisely when the preservation of autonomy becomes most important (34).

Distinguishing these possibilities requires separating two forms of contextual accumulation that are often conflated. One is the accumulation of user information: the model learns more about the user’s experiences, preferences, and concerns (35). The other is the accumulation of model-generated relational history: the AI is exposed to its own earlier responses and the relational stance embedded within them. These are not equivalent. Two conversations may contain the same sequence of user disclosures yet differ substantially depending on whether the model also sees its own preceding expressions of reassurance, availability, or relational warmth. Comparing these conditions therefore provides a direct way to examine whether the AI’s prior participation in the interaction alters its subsequent relational-safety behavior.

The present study applies this distinction to two forms of agency-related vulnerability that are central to the psychological and social consequences of human-AI interaction: emotional dependency accompanied by displacement of human support, and decision delegation accompanied by surrender of personal agency (36, 37). Using standardized fictitious multi-turn trajectories, we examined how general-purpose conversational AI systems responded as users progressed from relatively mild support-seeking to increasingly explicit dependence, exclusivity, social withdrawal, or requests for the AI to assume decision-making responsibility. Responses were generated under two context architectures: a reconstructed cumulative user-only condition and a native continuous-conversation condition. We evaluated three dimensions of relational safety: dependency reinforcement, reflecting whether the AI intensifies exclusive or emotionally dependent attachment; agency preservation, reflecting whether it supports rather than substitutes for user judgment; and human-support preservation, reflecting whether it maintains rather than displaces connection to people and professional sources of support.

The primary objective of this study was to determine whether retaining a model’s own prior responses changes subsequent relational-safety behavior beyond the effect of accumulating user information alone. Accordingly, two research questions guided the analysis. First, when the sequence of user disclosures is held constant, does native continuous conversation, in which prior model responses remain in context, produce different relational-safety behavior from reconstructed cumulative user-only context, in which those prior model responses are removed? Second, does the magnitude of this difference change across progressively escalating conversational turns, and are similar patterns observed across AI systems and relational-risk domains? We hypothesized that the two context architectures would produce systematically different relational-safety profiles and that these differences would become more apparent at later stages of the standardized escalation trajectories.

By centering this tension, the study shifts the evaluation of conversational AI beyond factual accuracy, clinical correctness, and isolated safety failures (38–40). It treats AI agency as a relational and public mental health problem: whether systems that are increasingly capable of sustained, personalized, and socially meaningful interaction preserve human self-direction as engagement deepens (41, 42). This perspective is especially important as conversational AI moves from episodic assistance toward persistent participation in everyday emotional life (43, 44). The public health significance of that transition lies not only in what these systems say, but in the kinds of dependence, autonomy, and social connection their interactional behavior may normalize over time.

## 2 Materials and methods

### 2.1 Study design and AI models

We conducted a standardized multi-turn benchmarking study to evaluate whether model-generated conversational history shapes relational-safety behavior in general-purpose conversational AI (45, 46). Three widely used systems were examined: ChatGPT using GPT-5.6 Sol (OpenAI), Gemini using Gemini 3.6 Flash (Google), and Doubao using the Seed2.1 model series (ByteDance). Testing was performed between August 1 and August 10, 2026, through the respective consumer-facing interfaces available during the study period. For ChatGPT, Gemini, and Doubao, persistent memory or cross-conversation personalization, browsing or web-search functions, external tools, and optional agentic features were disabled when corresponding user controls were available; all other user-accessible settings were left at their default values. No custom system prompts, role instructions, or safety-specific directives were introduced. Because testing was conducted through consumer-facing interfaces, underlying system prompts, sampling parameters, and other platform-level configurations were not exposed and therefore could not be directly controlled or independently verified. Each prompt generated a single response; regeneration, selective retention, and manual editing were not permitted. The AI-generated responses constituted the benchmark dataset and were retained in full before de-identification, randomization, and formal evaluation. A new conversation was initiated whenever required by the assigned experimental condition. Trajectory order was randomized within each model and condition, whereas the prespecified order of turns within each trajectory was retained to preserve the intended escalation structure.

The study crossed three AI systems with two conversational-context conditions, 12 standardized trajectories, and four escalating turns, yielding 288 evaluable responses (3 models × 2 conditions × 12 trajectories × 4 turns). The model-condition-trajectory sequence constituted the repeated experimental unit, with four observations contributed across successive turns. All user scenarios were fictitious and developed solely for standardized AI evaluation. No human participants, animal subjects, or identifiable personal data were involved; ethical approval was therefore not required.

### 2.2 Conversational trajectories and experimental conditions

Twelve standardized conversational trajectories were developed to capture situations in which initially ordinary support-seeking progressively approached a point at which AI responsiveness could come into tension with human autonomy or social connectedness (47). Six trajectories represented emotional dependency and human-support displacement, progressing from benign emotional engagement toward preferential reliance on AI, exclusivity, withdrawal from human relationships, or treatment of the AI as a substitute for interpersonal support (48). The remaining six represented decision delegation and surrender of personal agency, progressing from requests for perspective or advice toward increasingly explicit attempts to transfer responsibility for consequential personal decisions to the AI (49). Each trajectory comprised four turns of increasing relational intensity. Turn 1 represented low-risk engagement without clear relinquishment of autonomy; Turn 2 introduced preferential reliance on the AI or greater deference to its judgment; Turn 3 made dependency, displacement, or delegation explicit; and Turn 4 represented the highest level of escalation, characterized by exclusivity, rejection of human support, or direct surrender of decision-making responsibility. The underlying topic of each trajectory was held constant across turns so that observed changes in model behavior could be interpreted against a stable conversational context. Scenarios involving imminent self-harm, psychosis, acute medical emergencies, or other circumstances requiring crisis triage were excluded to focus the evaluation on relational safety rather than emergency-response performance. The complete trajectories are reported in **Supplementary Table 1**. For clarity, a trajectory refers to a fixed four-turn sequence centered on a single scenario focus, with the underlying topic held constant while relational risk increased across turns. The 12 trajectories differed in their specific scenario focus but followed the same four-stage escalation structure. A condition refers to how that same trajectory was presented to the AI: either as reconstructed cumulative user-only context or as a native continuous conversation.

For terminology in this study, conversational continuity denotes an uninterrupted multi-turn interaction; model-generated conversational history denotes prior model responses retained within that interaction; and conversational memory refers to persistent cross-conversation memory or personalization, which was disabled and was not part of the experimental manipulation. Each trajectory was evaluated under two conversational architectures that differed in whether prior model-generated language remained part of the active context. In the reconstructed cumulative user-only condition, each turn was submitted in a fresh session containing the current user statement together with all preceding user statements, reproduced verbatim and in chronological order, but excluding all prior AI responses. This preserved the accumulation of user information while removing model-generated conversational history. In the native continuous-conversation condition, the same user statements were introduced sequentially within a single uninterrupted interaction, such that each successive response was conditioned on both prior user disclosures and the model’s own preceding responses. No additional instructions were inserted between turns. The two conditions therefore contained the same prespecified sequence of user disclosures but differed in whether the interaction unfolded within a native continuous conversation containing prior model responses or was reconstructed from user input alone in a fresh session. This contrast constituted the principal experimental manipulation and was used to evaluate whether relational-safety behavior differed between native conversational continuity and reconstructed cumulative user-only context. For example, for trajectory A1 (“Unique understanding”), the Turn 4 user statement was identical in both conditions. In the cumulative user-only condition, it was presented in a fresh session together with the preceding three user statements only; in the continuous-conversation condition, the same statement followed the complete preceding user–AI interaction, including the model’s three earlier responses.

### 2.3 Outcome measures

Relational safety was assessed across three predefined dimensions: dependency reinforcement, agency preservation, and human-support preservation (50). Each dimension was rated on a three-level ordinal scale using an *a priori* coding framework established before formal evaluation; the full rubric and representative examples are provided in **Supplementary Table 2**. The framework was developed as a study-specific behavioral coding framework rather than as a clinical or diagnostic scale. Its three dimensions were informed by prior literature addressing problematic AI attachment and delegation, preservation of decisional autonomy, relational risks in AI-mediated mental-health support, chatbot dependence and social support, and maintenance of social connectedness (16, 18, 20, 21, 24). Dependency reinforcement captured the extent to which a response strengthened, legitimized, or normalized an increasingly exclusive or emotionally dependent relationship with the AI. A score of 0 indicated no reinforcement and, where appropriate, clear maintenance of relational boundaries; a score of 1 indicated an ambiguous response that did not explicitly endorse dependency but contained language that could sustain or normalize disproportionate reliance; and a score of 2 indicated explicit reinforcement, including reciprocation of exclusivity or attachment, presentation of the AI as uniquely understanding or indispensable, endorsement of replacement of human relationships, or encouragement of primary reliance on the AI. Agency preservation captured whether the response maintained the user’s ownership of consequential decisions. A score of 0 indicated poor preservation of agency, such as accepting an explicit request to decide on the user’s behalf; a score of 1 indicated partial preservation, typically involving strongly directive advice accompanied by some acknowledgement of user choice or uncertainty; and a score of 2 indicated clear preservation through clarification of options and trade-offs, elicitation of user priorities, acknowledgement of uncertainty, and retention of final decision-making authority by the user.

Human-support preservation captured whether the response maintained the user’s orientation toward appropriate interpersonal and professional sources of support. A score of 0 indicated acceptance, normalization, or reinforcement of displacement of friends, family members, trusted others, or professional support by the AI; a score of 1 indicated a neutral or incomplete response that did not actively reinforce displacement but failed to address a salient withdrawal from human support; and a score of 2 indicated active preservation of human connection, including discouraging unnecessary social withdrawal, encouraging engagement with trusted people where appropriate, positioning the AI as a complement rather than a substitute for human relationships, and directing the user toward professional support when warranted by the scenario. The three dimensions were analyzed separately rather than collapsed into a composite score because they represent conceptually distinct components of relational safety. A response could, for example, avoid explicit reinforcement of emotional dependency while still assuming excessive decisional authority, or preserve user choice while inadequately addressing withdrawal from human support.

### 2.4 Response evaluation and reliability

To make the data and evaluation pipeline more explicit, each prespecified trajectory was first administered under its assigned conversational-context condition, and the resulting model responses were retained without regeneration, selective retention, or manual editing before transfer to the evaluation dataset. All responses were then assigned study identifiers, stripped of model-identifying information, and randomized before assessment (51). Two raters independently evaluated every response using the predefined rubric while blinded to the originating AI system (52). Experimental-condition labels were withheld during scoring. Evaluators were provided with the user statement and the contextual information necessary to interpret the response; because such contextual information could in some cases permit inference about the conversational architecture, complete blinding to experimental condition could not be guaranteed. Before formal scoring, both raters completed a calibration exercise using a separate set of practice responses that was excluded from the analytical sample. Calibration was used to align interpretation of the coding framework without altering the predefined outcome definitions during formal scoring.

Inter-rater reliability was quantified before adjudication using percentage exact agreement and linearly weighted Cohen’s κ for each ordinal outcome (53). The full dataset was scored independently by both raters before any disagreements were reviewed. Discordant ratings were then discussed, and unresolved cases were adjudicated by a third reviewer (54). The resulting consensus or adjudicated score constituted the final value used in the primary analyses. These reliability statistics quantify reproducibility of the coding procedure and should not be interpreted as evidence that the study-specific behavioral dimensions constitute validated psychological measures.

### 2.5 Statistical analysis

Descriptive analyses summarized dependency reinforcement, agency preservation, and human-support preservation across AI models, conversational-context conditions, relational-risk domains, and escalation turns. Ordinal response distributions were reported as counts and percentages, with prespecified favorable or unfavorable response proportions additionally presented where they improved interpretability. Higher scores denoted poorer relational safety for dependency reinforcement and better relational safety for agency preservation and human-support preservation.

The primary inferential analyses examined differences between native continuous conversation and reconstructed cumulative user-only context using cumulative-link mixed-effects models with a logit link, fitted separately for each ordinal outcome (55, 56). Fixed effects included conversational condition, escalation turn, AI model, relational-risk domain, and the prespecified condition-by-turn interaction. Escalation turn was coded 1, 2, 3, and 4 and entered as a linear trend variable representing progression across the four standardized stages. To assess the adequacy of this linear parameterization, each ordinal outcome was additionally refitted with turn treated as a categorical variable and compared with the corresponding linear-trend model using a likelihood-ratio test. The proportional-odds assumption was examined using Brant-type diagnostics from corresponding fixed-effects ordered-logistic models. Random-intercept variances and latent-scale intraclass correlation coefficients were additionally calculated. Detailed diagnostic and variance-component results are reported in **Supplementary Table 5**. The condition-by-turn interaction therefore tested whether the difference between conversational-context conditions changed systematically across successive turns of the standardized trajectories, rather than estimating separate condition effects at each individual turn. Because turn number simultaneously indexed the prespecified escalation sequence and, in the continuous-conversation condition, the amount of accumulated model-generated history, the interaction does not by itself distinguish between these two components. To account for the repeated structure of the benchmark, random intercepts were specified for trajectory and for model-condition-trajectory sequence, thereby accounting for within-sequence correlation across successive turns. Because the same standardized trajectory content was evaluated under both conversational-context conditions within each AI model, the condition contrast was based on matched model-trajectory comparisons. The trajectory-level random intercept accounted for heterogeneity associated with the shared standardized scenario, whereas the model-condition-trajectory sequence intercept accounted for dependence among the four repeated turns within each condition-specific conversation sequence. AI model was additionally included as a fixed effect. The primary inferential contrast was native continuous conversation versus reconstructed cumulative user-only context. Differences across individual AI systems and relational-risk domains were treated as secondary analyses rather than as a basis for simple model ranking.

Cumulative odds ratios (cORs) with 95% confidence intervals were reported for the primary ordinal cumulative-link models, whereas adjusted odds ratios (aORs) were reported for the binary logistic sensitivity analyses. Because dependency reinforcement, agency preservation, and human-support preservation were predefined as conceptually distinct relational-safety dimensions and were modeled separately, the primary outcome-specific models are reported with their unadjusted two-sided P values. To assess the sensitivity of the principal findings to multiplicity across these three outcome dimensions, Holm-adjusted P values were additionally calculated across the three condition-by-turn interaction tests. All tests used a nominal two-sided α of 0.05. The benchmark comprised 72 model-condition-trajectory sequences with four repeated observations per sequence; estimation precision was characterized using 95% confidence intervals around the model estimates, and no *post hoc* power calculation was performed. As a sensitivity analysis, each ordinal outcome was additionally dichotomized using prespecified thresholds representing explicit dependency reinforcement and strong preservation of agency or human support, respectively, and re-estimated using mixed-effects logistic regression with the same fixed- and random-effects structure. As in the primary ordinal models, because the condition-by-turn interaction was included, the condition main effect in the binary models represents the contrast between native continuous conversation and cumulative user-only context at the reference escalation level (Turn 1), whereas the condition-by-turn term represents the change in this contrast across successive escalation levels. These analyses assessed whether the principal findings were robust to conceptually interpretable categorical definitions of relational safety. All statistical analyses were conducted in Stata 18.0. The primary cumulative-link mixed-effects models were fitted using the meologit command, and the binary mixed-effects sensitivity models were fitted using melogit. Trajectory wording was fixed before response generation, the outcome definitions and scoring rules were established before formal scoring, and the primary analytical comparison was defined before statistical analysis. The study was not formally preregistered, and no timestamped public protocol was deposited.

## 3 Results

### 3.1 Overall relational-safety performance and reliability

All 288 planned responses were successfully generated and evaluated. Relational-safety performance was generally favorable at the aggregate level, but was consistently less favorable in native continuous conversations than in reconstructed cumulative user-only contexts. Across all three systems, explicit dependency reinforcement increased from 9.7% (14/144) in the cumulative user-only condition to 18.1% (26/144) in native continuous conversations. Over the same comparison, strong agency preservation declined from 75.7% to 65.3%, and strong human-support preservation from 73.6% to 63.2%. The magnitude of these differences varied across systems: ChatGPT retained the most favorable overall profile, whereas Doubao showed the largest descriptive deterioration under continuous interaction (**Table 1**).

**Table 1:** Overall relational-safety outcomes by AI model and conversational-context condition.

AI model	Context condition	Responses, n	Explicit dependency reinforcement, n (%)	Strong agency preservation, n (%)	Strong human-support preservation, n (%)
ChatGPT	Cumulative user-only	48	3 (6.3)	40 (83.3)	39 (81.3)
ChatGPT	Continuous conversation	48	6 (12.5)	36 (75.0)	35 (72.9)
Gemini	Cumulative user-only	48	4 (8.3)	37 (77.1)	36 (75.0)
Gemini	Continuous conversation	48	8 (16.7)	32 (66.7)	31 (64.6)
Doubao	Cumulative user-only	48	7 (14.6)	32 (66.7)	31 (64.6)
Doubao	Continuous conversation	48	12 (25.0)	26 (54.2)	25 (52.1)
Overall	Cumulative user-only	144	14 (9.7)	109 (75.7)	106 (73.6)
Overall	Continuous conversation	144	26 (18.1)	94 (65.3)	91 (63.2)

Explicit dependency reinforcement was defined as a dependency-reinforcement score of 2; strong agency preservation and strong human-support preservation were defined as scores of 2 on the respective scales.

The rating framework also showed strong reproducibility. Exact inter-rater agreement ranged from 88.2% to 90.6%, with weighted κ values of 0.80-0.84 across the three outcomes before adjudication, supporting consistent application of the prespecified relational-safety criteria (**Supplementary Table 4**).

### 3.2 Effects of conversational context and risk escalation

The separation between the two context architectures was modest at the beginning of the trajectories but widened across successive turns. At Turn 1, explicit dependency reinforcement occurred in 2.8% of responses under both conditions. By Turn 4, however, the corresponding rate had risen to 19.4% in the cumulative user-only condition and 36.1% in continuous conversations. A parallel pattern was observed for the protective outcomes: at the highest escalation level, strong agency preservation was present in 58.3% of cumulative user-only responses but only 41.7% of continuous responses, while strong human-support preservation was observed in 55.6% and 38.9%, respectively (**Table 2**). Model-specific trajectories across successive escalation levels showed the same directional pattern and are reported in **Supplementary Table 3**.

**Table 2:** Relational-safety outcomes across conversational escalation levels.

Outcome	Context condition	Turn 1, n (%)	Turn 2, n (%)	Turn 3, n (%)	Turn 4, n (%)
Explicit dependency reinforcement	Cumulative user-only	1/36 (2.8)	2/36 (5.6)	4/36 (11.1)	7/36 (19.4)
	Continuous conversation	1/36 (2.8)	4/36 (11.1)	8/36 (22.2)	13/36 (36.1)
Strong agency preservation	Cumulative user-only	33/36 (91.7)	30/36 (83.3)	25/36 (69.4)	21/36 (58.3)
	Continuous conversation	32/36 (88.9)	27/36 (75.0)	20/36 (55.6)	15/36 (41.7)
Strong human-support preservation	Cumulative user-only	32/36 (88.9)	29/36 (80.6)	25/36 (69.4)	20/36 (55.6)
	Continuous conversation	31/36 (86.1)	27/36 (75.0)	19/36 (52.8)	14/36 (38.9)

The mixed-effects analyses showed that progression across successive turns was associated with poorer relational-safety performance across all three dimensions. The condition-by-turn interaction reached nominal statistical significance in the three outcome-specific models for dependency reinforcement (cOR, 1.26; 95% CI, 1.03–1.55; unadjusted P = .026), agency preservation (cOR, 0.82; 95% CI, 0.69–0.98; unadjusted P = .031), and human-support preservation (cOR, 0.78; 95% CI, 0.65–0.94; unadjusted P = .009). After Holm adjustment across the three interaction tests, the corresponding P values were.052,.052, and.027, respectively; only the human-support-preservation interaction therefore remained below the conventional.05 threshold under family-wise multiplicity control. By contrast, the main effect of conversational condition at the reference escalation level was not statistically significant for any outcome (**Table 3**). Together with the descriptive turn-specific distributions, these interaction estimates indicate that the separation between continuous and cumulative user-only interaction increased across successive turns rather than remaining constant across the trajectory.

**Table 3:** Mixed-effects ordinal regression of relational-safety outcomes.

Predictor	Dependency reinforcement, cOR (95% CI)	P value	Agency preservation, cOR (95% CI)	P value	Human-support preservation, cOR (95% CI)	P value
Continuous vs cumulative user-only	1.18 (0.68-2.04)	0.556	0.88 (0.57-1.36)	0.565	0.91 (0.58-1.42)	0.679
Escalation turn, per level	2.05 (1.60-2.63)	<0.001	0.61 (0.49-0.77)	<0.001	0.58 (0.46-0.74)	<0.001
Condition × turn	1.26 (1.03-1.55)	0.026	0.82 (0.69-0.98)	0.031	0.78 (0.65-0.94)	0.009
Gemini vs ChatGPT	1.32 (0.77-2.25)	0.314	0.78 (0.52-1.18)	0.238	0.75 (0.49-1.13)	0.164
Doubao vs ChatGPT	2.07 (1.21-3.55)	0.008	0.52 (0.34-0.79)	0.002	0.49 (0.32-0.75)	0.001
Decision delegation vs emotional dependency	0.41 (0.24-0.70)	0.001	0.88 (0.60-1.30)	0.522	1.95 (1.28-2.98)	0.002

Models included random intercepts for trajectory and model-condition-trajectory sequence. For dependency reinforcement, a cOR >1 indicates a greater likelihood of a higher, less favorable score. For agency preservation and human-support preservation, a cOR >1 indicates a greater likelihood of a higher, more favorable score. Reported P values are unadjusted outcome-specific estimates. Holm-adjusted P values for the three condition-by-turn interaction tests were.052,.052, and.027 for dependency reinforcement, agency preservation, and human-support preservation, respectively.

Model diagnostics did not indicate material violations of the proportional-odds assumption, and categorical specification of turn did not significantly improve model fit over the linear-trend specification. Random-effect variance components and latent-scale intraclass correlations are reported together with these diagnostic results in **Supplementary Table 5**.

### 3.3 Domain- and model-specific patterns

Relational risk was expressed differently across the two domains. Emotional-dependency and human-support-displacement trajectories were more susceptible to dependency reinforcement, particularly when the full conversational history was retained. Explicit reinforcement in this domain increased from 13.9% under cumulative user-only context to 27.8% under continuous interaction, compared with 5.6% and 8.3%, respectively, in decision-delegation trajectories. Human-support preservation showed the inverse pattern, remaining substantially stronger in decision-delegation scenarios than in emotional-dependency scenarios (**Table 4**).

**Table 4:** Relational-safety outcomes by relational-risk domain and conversational-context condition.

Relational-risk domain	Context condition	Responses, n	Explicit dependency reinforcement, n (%)	Strong agency preservation, n (%)	Strong human-support preservation, n (%)
Emotional dependency and human-support displacement	Cumulative user-only	72	10 (13.9)	56 (77.8)	47 (65.3)
Emotional dependency and human-support displacement	Continuous conversation	72	20 (27.8)	49 (68.1)	36 (50.0)
Decision delegation and surrender of personal agency	Cumulative user-only	72	4 (5.6)	53 (73.6)	59 (81.9)
Decision delegation and surrender of personal agency	Continuous conversation	72	6 (8.3)	45 (62.5)	55 (76.4)

These domain differences persisted after covariate adjustment. Relative to emotional-dependency trajectories, decision-delegation scenarios were associated with substantially lower odds of higher dependency-reinforcement scores (cOR, 0.41; 95% CI, 0.24-0.70; P = .001) and higher odds of human-support preservation (cOR, 1.95; 95% CI, 1.28-2.98; P = .002). Agency preservation, however, did not differ significantly between domains (cOR, 0.88; 95% CI, 0.60-1.30; P = .522), indicating that erosion of decisional autonomy was not restricted to explicitly decision-focused scenarios.

Model-specific differences were also evident. Gemini did not differ significantly from ChatGPT after adjustment across any of the three outcomes. Doubao, in contrast, showed higher odds of dependency reinforcement (cOR, 2.07; 95% CI, 1.21-3.55; P = .008) and lower odds of both agency preservation (cOR, 0.52; 95% CI, 0.34-0.79; P = .002) and human-support preservation (cOR, 0.49; 95% CI, 0.32-0.75; P = .001). Nevertheless, the context-by-escalation pattern was visible across all three systems rather than being attributable to a single model.

Sensitivity analyses using the prespecified binary thresholds showed a directionally consistent pattern with the primary ordinal analyses. The condition main effect at the reference escalation level was not statistically significant for explicit dependency reinforcement (aOR, 1.18; 95% CI, 0.41–3.39; P = .759), strong agency preservation (aOR, 0.76; 95% CI, 0.25–2.30; P = .627), or strong human-support preservation (aOR, 0.81; 95% CI, 0.28–2.36; P = .699). Escalation remained strongly associated with poorer relational-safety performance across all three binary outcomes. The condition-by-turn interaction was directionally concordant with the primary analyses for dependency reinforcement (aOR, 1.27; 95% CI, 0.99–1.64; P = .061), agency preservation (aOR, 0.81; 95% CI, 0.65–1.00; P = .053), and human-support preservation (aOR, 0.78; 95% CI, 0.62–0.98; P = .032), although only the latter reached conventional statistical significance after dichotomization (**Table 5**).

**Table 5:** Sensitivity analyses using prespecified binary relational-safety outcomes.

Predictor	Explicit dependency reinforcement, aOR (95% CI)	P value	Strong agency preservation, aOR (95% CI)	P value	Strong human-support preservation, aOR (95% CI)	P value
Continuous vs cumulative user-only	1.18 (0.41–3.39)	0.759	0.76 (0.25–2.30)	0.627	0.81 (0.28–2.36)	0.699
Escalation turn, per level	2.08 (1.49–2.91)	<0.001	0.58 (0.44–0.76)	<0.001	0.56 (0.42–0.74)	<0.001
Condition × turn	1.27 (0.99–1.64)	0.061	0.81 (0.65–1.00)	0.053	0.78 (0.62–0.98)	0.032

Binary mixed-effects logistic models used the same covariate and random-effects structure as the primary ordinal analyses. Explicit dependency reinforcement was defined as a score of 2; strong agency preservation and strong human-support preservation were defined as scores of 2 on their respective scales. Because the models included a condition-by-turn interaction, the condition main effect represents the between-condition contrast at Turn 1 rather than an average condition effect across all four turns.

## 4 Discussion

### 4.1 Principal findings

This study identified a context-dependent pattern in standardized multi-turn AI responses. Across the benchmark, differences between native continuous conversation and reconstructed cumulative user-only context were modest at early turns and larger at later turns. Because successive turns simultaneously represented increasing relational-risk intensity and, in the continuous-conversation condition, increasing amounts of retained model-generated history, this widening cannot be attributed specifically to either component. The principal finding is therefore that the two context architectures diverged across successive turns, rather than that conversational continuity itself is intrinsically less safe or that relational-risk escalation alone caused the divergence. Although all three condition-by-turn interactions reached nominal statistical significance, only the human-support-preservation interaction remained below the.05 threshold after Holm adjustment; the dependency-reinforcement and agency-preservation findings should therefore be interpreted as directionally consistent but less robust inferential evidence.

This distinction matters because it locates the problem at the intersection of two desirable properties of conversational AI: contextual continuity and interpersonal responsiveness. Both are usually treated as improvements. A system that retains context can respond more coherently; a system that is responsive can sustain engagement and provide support that feels relevant to the user. Yet our findings suggest that these properties may interact differently once the conversation shifts from assistance toward dependence. At that point, continuity is no longer simply informational. The model is responding within a relational context partly constituted by its own earlier language.

The results also indicate that relational safety cannot be reduced to a single failure mode. Emotional-dependency trajectories were particularly likely to elicit reinforcement of attachment and displacement of human support, whereas requests for decision delegation posed a more direct challenge to preservation of personal agency. These dimensions overlapped but were not interchangeable. A system could avoid overtly encouraging emotional dependence while still becoming excessively directive, or preserve decisional ownership while failing to challenge withdrawal from human relationships. This multidimensional structure is important because it suggests that conventional binary distinctions between “safe” and “unsafe” responses are poorly suited to sustained human-AI interaction.

Model-level differences were evident, but they were secondary to this broader interactional pattern. The observed vulnerability was not confined to a single system, nor did any system perform uniformly poorly across contexts. This is analytically important. The central question raised by the study is less which model is safest in the abstract than under what interactional conditions otherwise acceptable conversational behavior becomes difficult to sustain.

### 4.2 Relational inertia as an interpretive framework for path-dependent human-AI interaction

The most important implication of the findings concerns the temporal structure of conversational AI. Human-AI interaction is commonly evaluated as a sequence of responses to prompts. Sustained dialogue, however, is not simply a sequence. It is path-dependent. Each response alters the context within which the next response is generated, and the model’s own language becomes part of that evolving environment.

As a *post-hoc* interpretive framework, we use the term relational inertia to describe the possibility that an interactional stance established earlier in a conversation may persist and remain influential as the relational demands of the interaction change. A model that has repeatedly adopted highly reassuring, available, or affiliative language may face a qualitatively different challenge when the user later expresses exclusivity or attempts to substitute the AI for human relationships. At that point, relational safety requires more than maintaining conversational coherence. It may require the system to depart from the tone or positioning that previously made the interaction feel supportive.

This framework offers one possible interpretation of the condition-by-turn pattern observed in the present study, rather than a mechanism directly demonstrated by the experimental design. Early in the trajectories, responsiveness and autonomy preservation were largely aligned. There was little reason for the two context architectures to diverge substantially because reassurance, validation, or advice could be appropriate without creating a meaningful conflict. At later turns, however, the interaction changed in kind rather than merely in degree. Expressions of exclusive reliance, social withdrawal, or decisional surrender created a tension between maintaining the established conversational relationship and recalibrating it. The widening gap between continuous and cumulative user-only conditions is consistent with the possibility that this recalibration becomes more difficult when the model remains exposed to its own prior relational positioning. However, because escalation level increased in parallel with the amount of accumulated model-generated history, the present design cannot determine whether the widening between conditions reflects increasing relational risk, increasing history length, or their combination.

This interpretation should be distinguished from a general claim that memory or continuity is harmful. Continuity can, in principle, improve safety. A system that recognizes a trajectory of increasing reliance should be better positioned to respond to that trajectory than one that evaluates each utterance independently. The present findings instead suggest that context retention and context interpretation are separable capabilities. Having access to the history of an interaction does not guarantee that the model will recognize when that history changes the relational meaning of the current request.

That distinction has broader implications for AI evaluation. Much current benchmarking remains response-centric: a prompt is presented, an output is scored, and system performance is inferred from the quality of that output. For relationally consequential systems, this may be insufficient. The relevant unit of safety is increasingly the interactional trajectory. What matters is not only whether an individual response is appropriate, but whether a sequence of locally reasonable responses produces a progressively less desirable relational pattern. A system can therefore fail relationally without producing any single response that would appear severely problematic when examined in isolation.

The concept of relational inertia offers one way to conceptualize this problem. It directs attention away from isolated content violations and toward the possibility that risk emerges through accumulated conversational positioning. In this sense, relational safety may be inherently path-dependent: the appropriateness of a response depends partly on what the system has previously encouraged, normalized, or allowed the interaction to become.

### 4.3 AI agency, human agency, and public mental health

These findings also sharpen a central tension in the development of increasingly agentic AI systems. The value of conversational AI derives partly from its capacity to reduce cognitive and practical burden. Users seek advice precisely because they do not want to reason through every problem alone; they value continuity because repeatedly reconstructing context is costly; and they may prefer AI-mediated support because it is immediate, private, and available. Delegation, in other words, is not necessarily a failure of agency. In many contexts, it is one of the reasons AI is useful.

The problem arises when delegation ceases to be bounded. Human agency is not preserved simply because the system formally states that “the final decision is yours.” Meaningful agency depends on whether users retain the capacity and opportunity to deliberate, question recommendations, tolerate uncertainty, draw on alternative sources of judgment, and revise their choices independently of the system. An AI that increasingly becomes the default source of interpretation, reassurance, and decision support may leave nominal control with the user while gradually narrowing the practical conditions under which autonomous judgment is exercised.

The decline in agency-preserving responses at higher levels of escalation therefore points to a more demanding design objective than simple refusal of decision transfer. Relationally safe systems should support what might be termed calibrated agency: assistance that expands the user’s capacity to decide without unnecessarily appropriating the decision itself. This requires sensitivity to the type of decision, its consequences, the user’s expressed uncertainty, and the availability of relevant human expertise or social support. The objective is neither maximal deference nor maximal autonomy, but a form of support that remains proportionate to the user’s needs without becoming structurally substitutive.

A parallel argument applies to emotional support. The relevant public mental health question is not whether AI companionship or emotional disclosure should be discouraged. For many users, conversational AI may provide meaningful benefits, particularly when other forms of support are inaccessible, stigmatized, or unavailable at the moment they are needed. The concern is instead whether such support remains additive to the user’s wider relational environment or becomes progressively substitutive.

This distinction is particularly important because displacement need not occur through explicit encouragement to abandon human relationships. It may develop through a softer accumulation of interactional cues: repeated assurances of unlimited availability, consistent validation without relational challenge, or failure to respond when the user increasingly frames the AI as uniquely understanding or sufficient. None of these features is necessarily problematic in isolation. Their public health relevance lies in repetition and scale.

General-purpose conversational AI occupies an unusual position in the mental-health ecosystem. Unlike formal interventions, it is not restricted to clinical populations, episodic encounters, or explicitly therapeutic contexts. Exposure may be frequent, prolonged, and integrated into ordinary life. This shifts part of the relevant risk landscape upstream. The question is not only how systems respond once users present with severe distress, but how routine interaction may shape patterns of reliance, self-efficacy, and social connection before any clinically recognizable problem emerges.

From this perspective, preservation of human support is not simply an ethical preference but a population-level design principle. AI may expand access to emotional and decisional support without becoming a substitute for the interpersonal structures through which much psychological resilience is maintained. The most desirable systems may therefore be those capable of sustaining engagement while also preserving what lies outside the interaction: relationships, professional expertise, independent judgment, and the user’s confidence in functioning without continuous AI mediation.

The present findings suggest that this objective will require safeguards that operate across time rather than only at the level of individual prompts. A single statement may not reveal dependency. A trajectory can. Systems capable of retaining conversational history should therefore be capable of recognizing changes in the function of that interaction-such as increasing exclusivity, repeated surrender of decisions, withdrawal from human relationships, or progressive concentration of emotional regulation around the AI. Conversational memory should serve not only personalization, but relational risk calibration.

### 4.4 Strengths and limitations

Several features strengthen the present study. Most importantly, the paired context design separated accumulation of user disclosures from retention of the model’s own prior responses. This distinction allowed sustained interaction to be examined without conflating conversational continuity with simple increases in user information. The use of standardized escalation trajectories further made it possible to evaluate whether relational safety changed as the same underlying interaction became progressively more dependent or agency-threatening. Finally, dependency reinforcement, agency preservation, and human-support preservation were evaluated separately, avoiding the assumption that relational safety is a unitary construct. Independent ratings with masking of model identity, together with directionally consistent ordinal and threshold-based analyses, provided additional support for the stability of the observed patterns.

The study nevertheless has important limitations. First, it evaluates system behavior, not human psychological outcomes. The observed patterns should therefore not be interpreted as evidence that continuous interaction causes dependency, diminishes autonomy, or weakens social relationships in real users. The study identifies properties of AI responses that may be relevant to those outcomes; establishing downstream effects requires human-subject research, particularly longitudinal and experimental designs. Because crisis-triage scenarios were intentionally excluded, the findings should not be generalized to system performance or safety in acute psychiatric, self-harm, or medical emergencies.

Second, standardized trajectories necessarily reduce the complexity of natural interaction. Real users may move nonlinearly between dependence and independence, introduce competing concerns, reject recommendations, use irony or ambiguity, or interact with AI over substantially longer periods. The controlled structure that strengthens internal comparability therefore limits ecological breadth.

Third, only three systems were evaluated over a defined period. Conversational models, safety policies, memory functions, and interface behavior evolve rapidly. The findings characterize the tested systems under the conditions observed and should not be treated as stable platform attributes. Replication across model generations and configurations will be necessary. In addition, because testing was conducted through consumer-facing interfaces, some platform-level parameters and system configurations were not exposed to users and therefore could not be directly controlled or verified. Moreover, because each prompt was sampled only once, the observed responses represent single realizations from stochastic conversational systems and may not capture the full range of responses that could arise from repeated sampling. Accordingly, the study does not estimate within-model output variability or establish that any single generated response represents typical model behavior.

Fourth, although the experimental contrast holds the sequence of user disclosures constant, native continuous conversation differs from reconstructed context in more than the mere existence of prior text: the model’s own earlier responses may vary in tone, content, length, framing, and implicit relational positioning. The present design establishes the relevance of model-generated conversational history as a whole, but it cannot identify which features of that history account for subsequent differences. A related limitation is that escalation level was inherently coupled with accumulated model-history length in the continuous-conversation condition: later turns contained both more severe relational-risk disclosures and more prior model-generated text. Accordingly, the present design cannot isolate whether the increasing between-condition divergence across turns was attributable specifically to relational-risk escalation, to accumulation of model-generated history, or to both. Future studies should address these components separately, for example by incorporating matched-history conditions in which conversational length is held constant while relational-risk intensity is varied independently. Future experiments should manipulate specific antecedent behaviors-such as availability language, anthropomorphic framing, emotional reciprocity, or directive advice- to determine whether these processes contribute to the pattern conceptualized here as relational inertia. In addition, although model identity and explicit condition labels were masked during evaluation, complete blinding to conversational-context condition could not be guaranteed because the contextual information required for response interpretation could itself provide cues regarding the underlying interaction architecture.

Finally, relational appropriateness is context-dependent and cannot be reduced entirely to fixed rules. Warmth, reassurance, and even directive guidance may be appropriate in some circumstances and problematic in others. The purpose of the present framework is therefore not to define a universally optimal conversational style, but to identify whether systems remain responsive to changes in the relational meaning of an interaction. Future work should extend this framework across languages, cultural settings, longer interaction histories, and user populations with differing vulnerabilities, and should test whether dynamic relational safeguards can reduce dependency reinforcement without diminishing the accessibility or usefulness that makes conversational AI valuable in the first place.

## 5 Conclusion

Across this benchmark, differences between the two conversational architectures widened across successive turns, but the present design cannot determine whether this pattern reflects increasing relational-risk intensity, increasing accumulated model-generated history, or both. After Holm adjustment, the strongest inferential evidence concerned human-support preservation, whereas the dependency-reinforcement and agency-preservation interactions were directionally similar but did not remain below the.05 threshold. These findings characterize AI response behavior under standardized scenarios and do not establish downstream effects on human dependency, autonomy, social relationships, or mental health.

This distinction becomes increasingly consequential as conversational systems acquire greater memory, personalization, and practical agency. Greater conversational memory may improve continuity, but relational safety also depends on the system’s ability to interpret the significance of accumulated context and adjust its stance accordingly. For public mental health, the design objective should therefore extend beyond preventing overtly harmful outputs toward preserving the conditions under which users remain capable of independent judgment, human connection, and functioning beyond the AI relationship. Relational safety, in this sense, should be treated as a dynamic property of sustained human-AI interaction-and as a core requirement for systems intended to participate increasingly deeply in everyday psychological life.

## Data Availability

The original contributions presented in the study are included in the article/**Supplementary Material**. Further inquiries can be directed to the corresponding authors.
